# Locally Advanced Non Small Cell Lung Cancer: The Case for Radiation Dose De-escalation in the Management of the Mediastinum

**DOI:** 10.3389/fonc.2019.00283

**Published:** 2019-04-16

**Authors:** Viacheslav Soyfer, Benjamin W. Corn

**Affiliations:** ^1^Tel Aviv Medical Center, Tel Aviv University, Tel Aviv, Israel; ^2^Shaare Zedek Medical Center, Hebrew University of Jerusalem, Jerusalem, Israel

**Keywords:** lung, radiation, dose, de-escalation, radio-sensitivity

Outcomes for patients with locally-advanced Non Small Cell Lung Cancer (NSCLC) remain poor. In the context of definitive (as opposed to neoadjuvant) treatment, radiation oncologists have traditionally embraced dose escalation as a means to improve control of the primary tumor as well as draining nodal regions for this clinical problem. Yet we wonder: is it optimal—or even rational—to treat the primary and the mediastinal nodes to the same dose in these patients?

Enthusiasm for dose escalation was fueled by RTOG 73-01, a randomized trial that systematically explored incremental increases of dose which were correlated with improved intra-thoracic disease control in nearly linear fashion (1). Although the trial has acquired landmark status, it may not be germane for today's patients since radiation alone was used. It is conceivable that in the absence of chemotherapy, higher doses of radiation were needed. However, even when 60 Gy was delivered, a survival advantage did not emerge. At this juncture, standard management is predicated on a multi-modality approach.

The same cooperative group endeavored to push the dose envelope even further with an elegant trial design that harvested disappointing results (2). Specifically, RTOG 0617 revealed that 74 Gy was worse than 60 Gy. This negative finding appears to be, at least in part, due to excess toxicity related to incidental dose to normal mediastinal structures (e.g., esophagus and heart). In a secondary analysis of 0617, Chun et al. determined that intensity modulated radiation therapy produces less heart exposure when compared to 3-D techniques (3). Nevertheless, in a pooled analysis of 112 patients with stage III NSCLC treated on six separate prospective trials, Wang et al. (4) emphasized that cardiac events are multi-factorial in nature but that among those factors, radiation dose to the heart must be respected when considering cardiac risk. Wang et al. underscore that the latency to cardiac effects may be unexpectedly short in patients irradiated for NSCLC (in comparison to those receiving irradiation for breast cancer and Hodgkin's lymphoma) due to the high prevalence of smoking histories and pre-existing cardiac disease. As such, Wang et al. advocate minimizing doses to the heart in patients with stage III NSCLC. This therefore raises the question of the logic of applying higher doses to both the primary site and regional nodes.

There are several reports suggesting that involved mediastinal nodes can be sterilized with definitive conventional doses of irradiation. For example, the mediastinal nodes appeared to be cleared in 63% (27/43) of patients treated with ≈61.2 Gy + concurrent chemotherapy in RTOG 0229 (5). In a subsequent trial carried out by NRG Oncology (6), patients with locally-advanced NSCLC who were neoadjuvantly treated with carboplatin, paclitaxel and 60 Gy demonstrated mediastinal nodal sterilization of 68%, however, the trial was terminated prematurely due to grade 4–5 toxicities in excess of 15%. It could not be established that the high mortality and toxicity rate were related to the dose of preoperative radiation or other *post-operative* effects. The authors further cautioned that the value of 60 Gy (vs. 45 Gy) has not been established in a phase III trial and should not be considered a standard of care.

There is a growing awareness of the dangers arising from irradiating adjacent thoracic organs that were previously not incorporated into risk-benefit models (e.g., esophagus, cardiac tissues, non-involved pulmonary parenchyma) when the lung and mediastinum are treated (7). Moreover, cardiac irradiation has been implicated in immunosuppression, as manifest by an increased neutrophil-to-lymphocyte ratio, which could indirectly worsen tumor control (8). These aggregate fears were accentuated when, in reporting their experience with cisplatin and high dose radiotherapy (approaching 66 Gy), Dieleman et al. (9) found that the gross tumor volume (GTV) of mediastinal lymph nodes emerged as the best predictor of treatment toxicity. Specifically, those author described mortality from mediastinal complications such as esophageal ulcer, mediastinal hemorrhage, and heart failure which were associated with lymph node GTV thus underscoring the importance of carefully attending to dose and volume issues of those lymph nodes.

Finally, two understated reports from northern Europe deserve attention. Van den Bosch et al. (10), in describing the results of treating 75 patients with locally-advanced NSCLC, observed that lymph node control can be achieved at *lower* radiation doses than needed for primary tumor control. Specifically, in a meticulous regression analysis, those authors could not identify a dose effect when assessing the endpoint of nodal relapse. What's more, in an analysis of 331 NSCLC patients treated with definitive radiation therapy in Denmark, Schytte et al. noted that 93 patients had loco-regional failure only. Of these, the majority (68 patients) had intra-pulmonary failure only, one patient had mediastinal failure only and 24 had mediastinal as well as pulmonary failure (11). The authors concluded that to improve locoregional control, attention must mostly be paid to the intrapulmonary tumor volume. Similar observations—regarding an uncoupling of radiosensitivity between primary disease and draining lymph nodes—were made in colorectal cancer (12) as well as head and neck tumors (13).

Currently, radiation dose de-escalation is advocated as a strategy for relatively rare entities (e.g., p16-positive oropharyngeal cancer, lymphoma, testicular tumors, pediatric malignancies). In addition, there are several scenarios (e.g., tumors of the head and neck, pelvis, etc.) where differential doses are prescribed for primary tumors and the draining nodes, respectively. We acknowledge the relatively high rates of mediastinal sterilization in the context of locally-advanced NSCLC. However, thorough follow-up and the use of patient reported outcome measures may continue to reveal potential morbidity associated with dose escalation to the mediastinum (whose nodes often constitute a challenging GTV after meticulous contouring has been completed).

To be sure, there has been renewed interest in this topic in the wake of the exciting results published by the authors of the PACIFIC trial (14, 15). The incorporation of immunotherapy in management of locally-advanced NSCLC significantly improves progression free and overall survival). The effect of the addition of Durvalumab to definitive chemo-radiation in stage 3 NSCLC has been shown to be independent of radiation dose. The hazard ratio (HR) for the overall survival in the Durvalumab and placebo groups for the randomization within or longer than 14 days was 0.42 (CI 0.27–0.67) vs. 0.81 (CI 0.0.62–1.06), respectively (14). Theoretically, lower irradiation dose and esophageal exposure might decrease acute toxicity and shorten the recovery period, which in turn could allow expedited initiation of immunotherapy.

Given these considerations and the likelihood that differential radiosensitivity exists as a function of underlying biologic differences such as variance in mitotic rates, necrotic components or levels of oxygenation (16), this may be the time to design a cluster of trials that explore dose de-intensification of the mediastinal nodes. A plausible design might be a randomized phase II comparison of 60 Gy vs. 56 Gy (all treatments delivered via conventional fractionation) to the mediastinum that seeks to establish non-inferiority vis-à-vis the endpoint of regional control at 2 years (Such a trial could simultaneously explore other questions such as dose escalation to primary disease; however, this is not the subject of the current report.). In this classical randomized two-arm non-inferiority design that compares a novel approach to a standard of care, we assume that the mediastinal recurrence in the respective arms is ~30% with a non-inferiority margin of 5%. Such a design would behoove the recruitment of ~1,100 patients in each group (calculations performed with SAS 9.4 software Proc POWER). Such a proposal could only be deployed in an organized multi-institutional framework. In other words, large consortiums (e.g., NRG Oncology, EORTC) would need to determine the prioritization of such a concept.

Alternatively a straightforward “estimation study” could be launched which simply aims to evaluate the frequency of nodal recurrence at the moderately lower mediastinal dose (56 Gy) which would be compared to literature-based standards. In so doing, ~400 patients would need to be enrolled to obtain 95% two-sided confidence interval of length 0.1. Although less conclusive in nature, the smaller sample would eliminate the dependence on enlisting the large cooperative groups with their attendant bureaucratic hurdles. Instead, patients could be drawn from several high-volume centers that are experienced in the treatment of lung cancer. If interest exists within the clinical research community to pursue such an idea, we might learn that it is possible to improve the therapeutic ratio of one of the most commonly occurring cancers in men and women.

## Author Contributions

VS and BC had an equal contribution in design and writing the manuscript.

### Conflict of Interest Statement

The authors declare that the research was conducted in the absence of any commercial or financial relationships that could be construed as a potential conflict of interest.
